# The number of beds occupied is an independent risk factor for discharge of trauma patients

**DOI:** 10.1097/MD.0000000000031024

**Published:** 2022-10-07

**Authors:** Sascha Halvachizadeh, Daniel Leibovitz, Leonhard Held, Kai Oliver Jensen, Hans-Christoph Pape, Dominik Muller, Valentin Neuhaus

**Affiliations:** a University Hospital Zurich, Department of Trauma, Zurich, Switzerland; b University of Zurich, Faculty of Medicine, Zürich, Switzerland; c University of Zurich, Epidemiology, Biostatistics and Prevention Institute, Zurich, Switzerland; d Cantonal Hospital Thurgau, Frauenfeld, Department of Surgery, Frauenfeld, Switzerland.

## Abstract

Reducing the burden of limited capacity on medical practitioners and public health systems requires a time-dependent characterization of hospitalization rates, such that inferences can be drawn about the underlying causes for hospitalization and patient discharge. The aim of this study was to analyze non-medical risk factors that lead to the discharge of trauma patients. This retrospective cohort study includes trauma patients who were treated in Switzerland between 2011 and 2018. The national Swiss database for quality assurance in surgery (AQC) was reviewed for trauma diagnoses according to the ICD-10 code. Non-medical risk factors include seasonal changes, daily changes, holidays, and number of beds occupied by trauma patients across Switzerland. Individual patient information was aggregated into counts per day of total patients, as well as counts per day of levels of each categorical variable of interest. The ARIMA-modeling was utilized to model the number of discharges per day as a function of auto aggressive function of all previously mentioned risk factors.

This study includes 226,708 patients, 118,059 male (age 48.18, standard deviation (SD) 22.34 years) and 108,649 female (age 62.57, SD 22.89 years) trauma patients. The mean length of stay was 7.16 (SD 14.84) days and most patients were discharged home (n = 168,582, 74.8%). A weekly and yearly seasonality trend can be observed in admission trends. The mean number of occupied trauma beds ranges from 3700 to 4000 per day. The number of occupied beds increases on weekdays and decreases on holidays. The number of occupied beds is a positive, independent risk factor for discharge in trauma patients; as the number of occupied beds increases at any given time, so does the risk for discharge.

The number of beds occupied represents an independent non-medical risk factor for discharge. Capacity determines triage of hospitalized patients and therefore might increase the risk of premature discharge.

## 1. Introduction

Triage of patients is a multidisciplinary challenge that is performed prior, during, and after hospital admission of patients: During mass casualty the primary triage strategy bases on the available resources of the trauma center.^[1,2]^ Numerous investigations have assessed algorithms to support decision making during prehospital triage.^[3,4]^ Even during routine pre-hospital triage, algorithms have been developed that are subject to constant optimization.^[5]^ The brief physician-patient interaction during emergency triage led to a sensitivity of 74%, and specificity of 84% for predicting admission.^[6]^ Miss-triage might therefore adversely affect the quality of the individual trauma care.^[7]^

During hospitalization, patients are constantly evaluated for readiness for hospital discharge. The lack of high quality evidence for timing, admission, discharge and triage process leads to the development of individual protocols and Grade 2 recommendations.^[3]^

Inappropriate discharge leads to early readmission^[8]^ and is associated with impaired outcome.^[9]^ Medical factors that promote early discharge with comparable results to routine discharge include younger age, and lower rate of comorbidities.^[10]^ Non-medical factors that delay discharge include durable administrative processes, and limited discharge times.^[11]^ Further, planning of discharge location might prolong hospital stay. This might include a requirement of a nursing home, or a rehabilitation clinic. Patient occupies beds until an admission time to an appropriate follow-up unit has been defined. Inappropriate triage and premature discharge are associated with increased morbidity and mortality.^[12]^ Evidence of the effect of bed occupancy on triage is scarce. Therefore, the goal of this study was to investigate the effect of bed-occupancy and seasonality on the discharge of trauma patients to answer the following research question: Are seasonality and the number of occupied beds independent risk factors for discharge in trauma patients?

## 2. Methods

The reporting of this retrospective cohort study strictly follows the Strengthening the Reporting of Observational Studies in Epidemiology (STROBE) Statement.^[13]^ The data is gathered from an anonymized national database. It is generated from a quality management initiative and the local ethical committee has waived the necessity of approval.

### 2.1. AQC database

This study utilizes a prospective surgical database (Arbeitsgemeinschaft für Qualitätssicherung, AQC) as described previously.^[14]^ This database is part of the mandatory quality control, surgical education and scientific analysis plan where more than seventy surgical departments in Switzerland document their inpatient surgical cases.^[15,16]^ The database includes date, time and type of surgery (elective vs. emergent and inpatient vs. outpatient), Swiss surgical procedure classification (CHOP) codes,^[17]^ the surgeons experience level (resident, fellow, attending or chief of surgery), and intra- or postoperative complications. The database further includes demographic information, the American society of Anesthesiologists (ASA) score,^[18]^ and length of stay.

### 2.2. Quality of data and missing values

The data quality depends on the data entry. Some restrictions are provided by the database to avoid unrealistic input errors. Some data fields are required in order to complete the form to improve completeness of data. Contribution to the AQC database is not mandatory, meaning that AQC data could be subject to selection bias. Data is not labeled by region, which obscures any potential geographical relationships (e.g., do increased trauma patients in a given canton have a stronger effect on nearby cantons than far-away cantons?). It is possible that some patients are observed more than once in the AQC database, meaning that their observations are correlated. Given the small proportion of patients expected to be seen twice, this possibility is ignored.

### 2.3. Study population

This study includes all trauma patients who were hospitalized in a Swiss surgical institution between 2011 and 2018. The trauma patients were extracted from the database by filtering the ICD-10 code for the main diagnosis that begins with the letter S or T, representing injury related diagnoses.

### 2.4. Variables and definitions

The primary outcome of this study was patient discharge as documented on the AQC database. The exposure of interest was the number of occupied beds. Effect modifiers included the patient’s demographics, surgical diagnosis and comorbidities, ASA, seasonal, weekly and daily timing of hospitalization and hospital discharge. Seasonality was assessed with a special focus on Swiss national holidays that included May 1st, Swiss national day (August 1st), and Christmas Eve (December 24th).

### 2.5. Statistical methods

For exploratory purposes, admission rates were first decomposed by the ETS (Error Trend Seasonality) method in order to visualize weekly, yearly and overall seasonality and trend. Raw admission rates were also compiled into the number of occupied beds by day, number of discharges by day, and proportion (or percentage) of discharges by day. Each of these four time series (raw admissions by day, occupied beds by day, discharges by day, proportion discharge by day) was modeled with an Auto-regressive integrated moving average (ARIMA) model. Each ARIMA model included has parameters *p*, *q*, and *d* selected by minimizing over the AIC.^[19]^ Each ARIMA model additionally includes weekdays, holidays, and a variable number of Fourier terms as external covariates. The Fourier terms are added to adjust for seasonality, and the degree of the Fourier series is again selected by minimizing over the AIC of each candidate model. Coefficients from all ARIMA models are shown without the values of the Fourier terms; coefficient tables including Fourier terms are included.

## 3. Results

This study includes 227,019 patients at a mean age of 55.06 (SD 23.73) years and 47.9% female. The majority of patients were classified as ASA 1 (42.4%). The overall mortality rate was 1.6%. The majority of patients were discharged home after hospitalization (74.8%). The mean duration of hospitalization was 7.16 (SD 14.84) days (Table 1).

**Table 1:** Descriptive statistics of patient data.

n	227,019
Male sex, n (%)	118,059 (52.1)
Female sex, n (%)	108,649 (47.9)
Age [years], mean (SD)	55.06 (23.73)
**ASA medical risk, n (%**)	
Level 1	95673 (42.4)
Level 2	88678 (39.3)
Level 3	37797 (16.8)
Level 4	3004 (1.3)
Level 5	237 (0.1)
Level 6	7 (0.0)
**Discharge location, n (%**)	
Death	3566 (1.6)
Home	168582 (74.8)
Hospital or care home	9067 (4.0)
Retirement home	7548 (3.3)
Psychiatric dept	1603 (0.7)
Rehabilitation dept	17458 (7.7)
Hospital or natal dept.	6063 (2.7)
Correctional facility	328 (0.1)
Neonatology dept.	9 (0.0)
Different dept.	1889 (0.8)
Hospice	25 (0.0)
Psychiatric dept.	40 (0.0)
Rehabilitation dept.	209 (0.1)
Acute dept.	83 (0.0)
Other	1354 (0.6)
Unknown	7556 (3.4)
Length of stay [days], mean (SD)	7.16 (14.84)

ASA = American Society of Anaesthesiology, dept. = department, n = numbers, SD = standard deviation.

**Figure 1.**
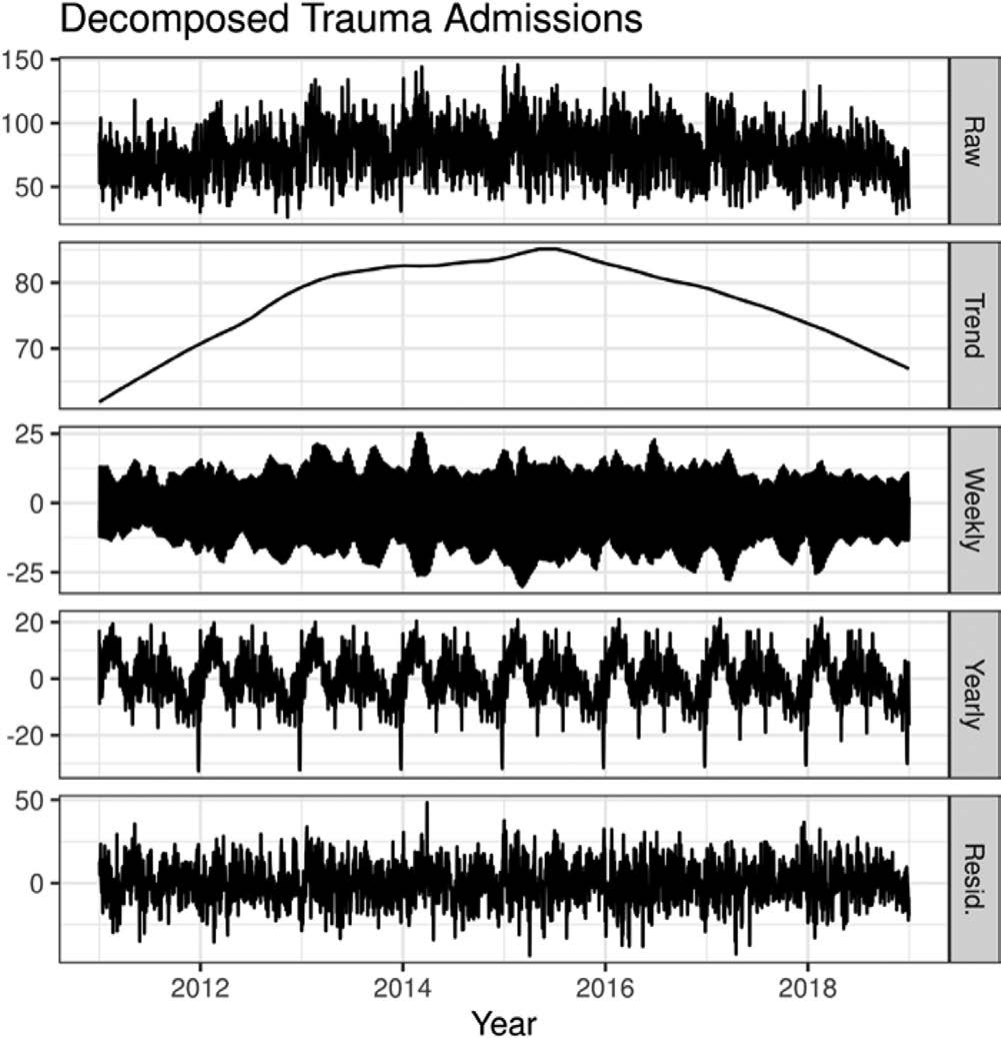
Swiss trauma admissions are decomposed into the long-term trend, weekly seasonality, yearly seasonality, and residuals (or noise).

The decomposed trauma admissions show a long-term trend, as well as yearly and seasonal cycles (Fig. 1). Most trauma patients are admitted during the early third of the year, with a peak in mid-February.

The mean number of occupied trauma beds in Switzerland is 3823.74, SD 81.99, range 3276 to 4042. Most beds are occupied during the months of February and August, and the lowest occupation rate was observed to be immediately after Christmas Eve.

**Figure 2.**
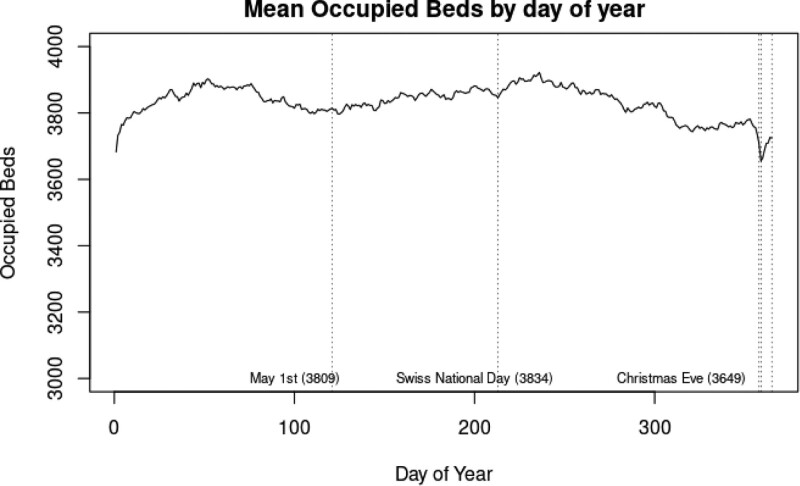
Mean number of beds occupied by trauma patients in Switzerland, by day of year. This time-series is much less volatile than the admission counts seen in Figure 1. Since the number of occupied beds is partly under human control. It is nonetheless still dependent on time, and appears to have a trend across the calendar year.

The number of occupied beds is less volatile when compared with the admission rates and shows a seasonal cycle. On Christmas Eve the mean number of beds occupied were the lowest during the year (n = 3649) (Fig. 2).

**Figure 3.**
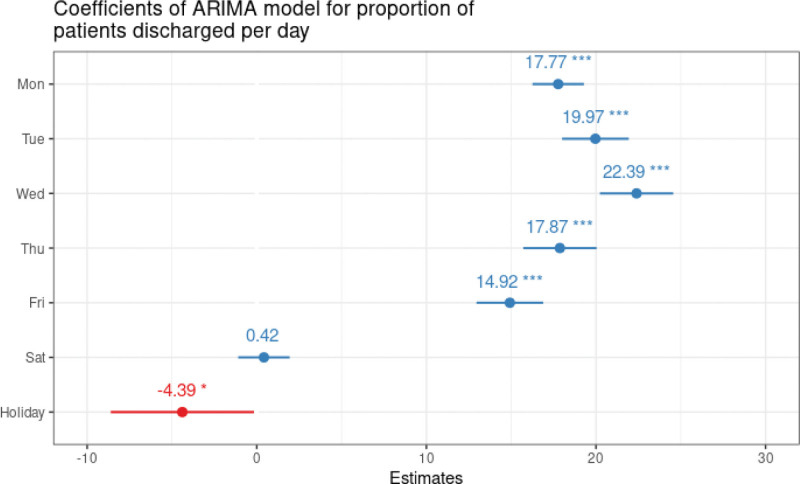
Coefficients are drawn from an ARIMA model of number of occupied beds with harmonic seasonal terms, and with Weekday and Holiday as external variables.

After correction for effect modifiers, the numbers of occupied beds were comparable on weekends. However, during the week, the number of occupied beds increases significantly with the peak reached on Wednesdays with 22.4 (95%CI 22.0‐24.0) beds more occupied when compared with Sundays. On Holiday 4.4 (95%CI -8.7‐-0.28) beds are less occupied when compared to Sunday (Fig. 3).

**Figure 4.**
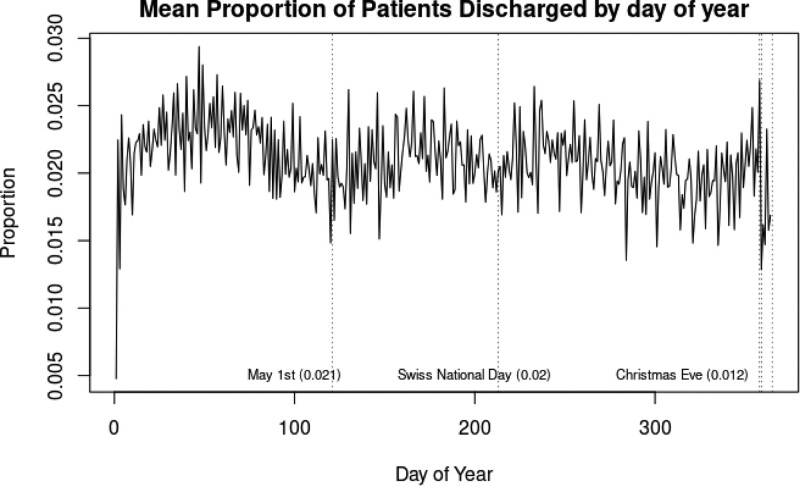
Mean proportion of current trauma inpatients in Switzerland discharged, by day of year. If the release of each patient didn’t depend on how many other beds are currently occupied, we would expect this graph to be pure noise. Instead, we see a trend over the course of the year, indicating that patient discharge decisions are dependent on the number of currently occupied beds.

**Figure 5.**
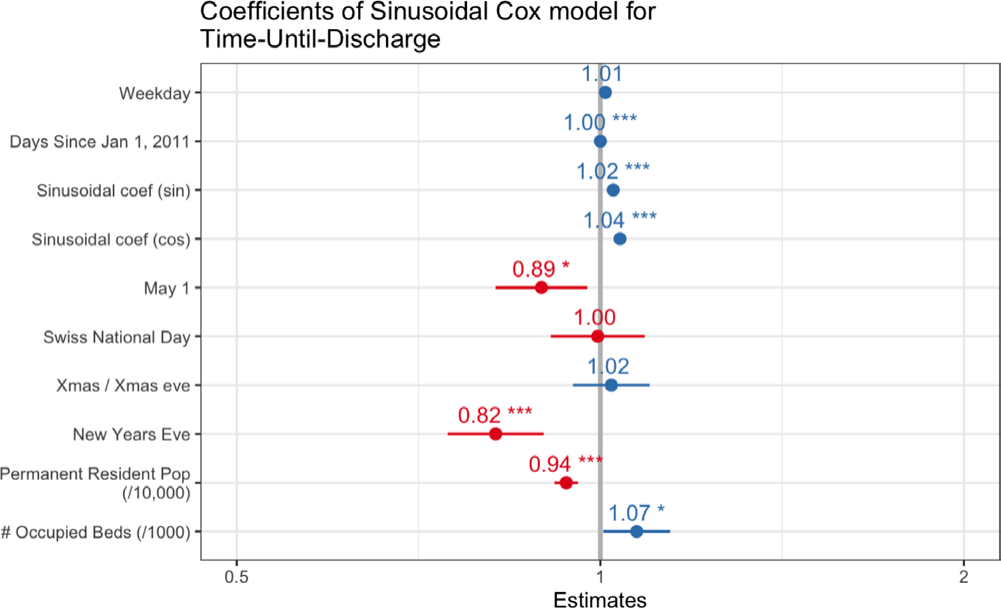
A Cox Sinusoidal regression: given the conditions on the day a patient was admitted, was the patient likely to have a longer or shorter stay in hospital? Red coefficients indicate a longer stay, blue coefficients indicate a shorter stay. An increase in Perm Resident Population predicts a longer stay and an increase in number of occupied beds a shorter stay. We divided the perm resident population by 10,000 and the beds occupied by 1000 to increase the size of the coefficients. This means that the effect, for beds for example, is the effect of an increase in 1000 beds occupied instead of the effect of an increase in 1 bed occupied.

The mean proportion of patients discharged over the year was 2.06% (SD 0.62%) and shows a yearly and a weekly cycle (Fig. 4). The proportion ranges from 0.05% to 5.21%. The mean proportion of patients discharged was highest in February, and lowest immediately after Christmas. At the major holidays the mean proportions of discharges were smallest. The duration of stay is further depending on an increase in Perm resident population, and on an increase in the number of occupied beds (Fig. 5). The weekdays have an independent positive effect on the proportion of patients discharged, whereas during the weekends and the holidays this observed effect is contrary. The number of occupied beds represents a significant and independent risk factor for patients discharge (Mean OR per singular bed 1.00095, 95%CI 1.00077‐1.0011) (Table 2).

**Table 2:** Point and interval estimates of ARIMA model for proportion of patients discharged per day.

	Mean	Lower CI (95%)	Upper CI (95%)
Sunday	1	Reference	
Monday	0.15	0.11	0.2
Tuesday	0.12	0.08	0.16
Wednesday	0.2	0.16	0.24
Thursday	0.19	0.15	0.23
Friday	0.12	0.08	0.16
Saturday	–0.13	–0.17	–0.08
Holiday	0.035	–0.052	0.12
Occupied beds	0.00096	0.00077	0.0012

ARIMA = autoregressive integrated moving average, CI = confidence interval.

## 4. Discussion

A certain seasonal cyclic pattern of trauma admissions has previously been described.^[20,21]^ There is still a lack of evidence on the effect of the number of occupied beds on discharge. This study aimed to investigate the effect of seasonal admission and number of occupied beds on the discharge of trauma patients and found the following points:

1. The weekly trend includes a “fill-up” of beds during the weekdays, the yearly trend a “fill-up” during February, and August.
2. The proportion of patients discharged depends on seasonal and weekly changes.
3. The number of occupied beds is an independent risk factor for discharge.

Compared with elective orthopedics and interventions, admission of trauma patients is usually more volatile. During weekends and nighttime most surgical interventions are emergency surgeries including temporal stabilization.^[14,22]^ During the weekday the definitive surgical procedures are performed and elective surgeries are planned.^[14]^ This leads to a “fill-up” of trauma patients over the week and to increased rate of discharge towards the end of the week. Timing of discharge after trauma surgery is a multifactorial challenge that includes consideration of mobilization status of the patients,^[23]^ requirement and intensity of postoperative rehabilitation,^[24]^ and requirement of special medical care or nursing facilities.^[25]^ The association of trauma admission and number of discharges appears reasonable with a certain seasonal variation. During the winter season the incidence of geriatric hip fractures increases by 8% when compared with summer months.^[26]^ The incidence of pediatric fractures increases in the summer months.^[27]^ These seasonal changes might be therefore mirrored in our results that indicate a seasonal and weekly association of patient discharge. Despite the seasonal variability, and the volatile admission of trauma patients, the number of beds represents an independent risk factor for patients discharge. According to our data, the discharge of one trauma patient is influenced by the occupancy of the bed next to that patient.

The discharge rate is further limited by administrative factors. Increased occupancies in rehabilitation centers and clinics postpone discharge of trauma patients.^[28,29]^ The discharge is more likely to succeed when procedures, and discharge timing can be planned and patient support following discharge have been arranged. A potential over-hospitalization should be mentioned in this discussion. Current efforts aim to minimize hospitalization and to utilize outpatient procedures as often as possible based on well-known advantages and disadvantages. However, these interventions have been mainly investigated in elective orthopedic surgery.^[30]^ The translation of these results into trauma care requires more detailed investigations.

This study is based on a national database that covers trauma patients independent of local geographic. The major limitation of this study was the lack of documentation of the reason for delayed discharge, or the reason for hospitalization. We however took all documented factors including seasonality into our prediction model and still found that the occupancy of beds represents an independent predictor for discharge. Further, the retrospective study design is subject to well-known limitations. We believe, however, that the nation wide data base provides enough data that allows a profound conclusion. One should take into consideration the weekday or holiday effect. These are the effects of those indicators when the patient was admitted. It should not matter if a patient was admitted on a weekday but discharged on a weekend. This represents one limitation of the ARIMA model.

## 5. Conclusion

Trauma admission is volatile, however, certain seasonality can be observed. Bed occupancy is an independent non-medical risk factor for patient discharge. Triage and hospital discharge are challenging processes and should primarily depend on the medical needs of the patients.

## Author contributions

**Conceptualization:** Daniel Leibovitz, Sascha Halvachizadeh, Valentin Neuhaus.

**Data curation:** Dominik Muller, Leonhard Held, Kai Oliver Jensen, Sascha Halvachizadeh, Valentin Neuhaus.

**Formal analysis:** Daniel Leibovitz, Leonhard Held, Valentin Neuhaus.

**Investigation:** Daniel Leibovitz, Hans-Christoph Pape, Kai Oliver Jensen, Sascha Halvachizadeh.

**Methodology:** Dominik Muller, Sascha Halvachizadeh.

**Project administration:** Hans-Christoph Pape, Valentin Neuhaus.

**Software:** Daniel Leibovitz, Sascha Halvachizadeh.

**Supervision:** Leonhard Held, Valentin Neuhaus.

**Validation:** Daniel Leibovitz, Dominik Muller, Hans-Christoph Pape, Leonhard Held, Valentin Neuhaus.

**Writing – original draft:** Daniel Leibovitz, Sascha Halvachizadeh.

**Writing – review & editing:** Daniel Leibovitz, Dominik Muller, Hans-Christoph Pape, Kai Oliver Jensen, Sascha Halvachizadeh, Valentin Neuhaus.
